# Predicting Performance of Lightweight Concrete with Granulated Expanded Glass and Ash Aggregate by Means of Using Artificial Neural Networks

**DOI:** 10.3390/ma12122002

**Published:** 2019-06-22

**Authors:** Marzena Kurpinska, Leszek Kułak

**Affiliations:** 1 Faculty of Civil and Environmental Engineering, Gdansk University of Technology, 80-233 Gdansk, Poland; 2 Faculty Applied Physics and Mathematics, Gdansk University of Technology, 80-233 Gdansk, Poland; kulak@mif.pg.gda.pl

**Keywords:** building, lightweight concrete, granulated expanded glass aggregate, artificial neural networks, prediction properties, granulated ash aggregate, artificial neural networks

## Abstract

Lightweight concrete (LWC) is a group of cement composites of the defined physical, mechanical, and chemical performance. The methods of designing the composition of LWC with the assumed density and compressive strength are used most commonly. The purpose of using LWC is the reduction of the structure’s weight, as well as the reduction of thermal conductivity index. The highest possible strength, durability and low thermal conductivity of construction materials are important factors and reasons for this field’s development, which lies largely in modification of materials’ composition. Higher requirements for construction materials are related to activities aiming at environment protection. The purpose of the restrictions is the reduction of energy consumption and, as a result, the reduction of CO_2_ emission. To limit the scope of time-consuming and often high-cost laboratory works necessary to calibrate models used in the test methods, it is possible to apply Artificial Neural Networks (ANN) to predict any of the concrete properties. The aim of this study is to demonstrate the applicability of this tool for solving the problems, related to establishing the relation between the choice of type and quantity of lightweight aggregates and the porosity, bulk density and compressive strength of LWC. For the tests porous lightweight Granulated Expanded Glass Aggregate (GEGA) and Granulated Ash Aggregate (GAA) have been used.

## 1. Introduction

For several decades, we have been observing steadily growing popularity of cement composites. This trend depends most of all on continuous research, aimed at environment protection and improving basic properties of the materials, as well as on giving them new, innovative features. One of the many examples of that is lightweight concrete (LWC) containing lightweight artificial aggregates. Its enormous advantage is that the components for the production of artificial aggregates are recycled or are waste materials [1,2,3,4,5,6]. 

Lightweight cement composites, in particular, LWC is one of the most important groups of materials, used in the construction industry. This is due to its main advantages, such as: possibility to obtain any shape of the element, good physical, mechanical and chemical properties, as well as good insulating properties [7,8,9,10]. In order to determine the impact of individual components on the properties of the LWC, various design methods are used, namely, experimental and analytical-experimental. The suitability of these methods for computer applications is also relevant [11,12,13,14,15]. It should be, however, emphasised that each method of designing of the composition of concrete mixture must be verified experimentally. 

Necessary tests, aiming at calibration of the proposed models, are being carried out, taking into account quality and quantity parameters of the components [16,17,18,19,20]. The tests are usually time-consuming and expensive. What is more, they must be repeated in case of each significant change of the type or quality of even one concrete component. Therefore, it is reasonable to use such models, which correlate components and concrete properties so as to minimise laboratory works. 

The application of artificial neural networks (ANN) to predict important parameters of concrete, including LWC, can be an example here [21,22,23,24]. The main purpose of the work is to present computer techniques in the form of ANNs, which will be used to predict compressive strength, porosity and bulk density of LWC, depending on the selection of the quantitative and qualitative composition of lightweight aggregates (LWA). ANN can be used for predicting output data, based on a defined input dataset [25,26]. 

The advantage of using computer techniques, involving ANN, to solve the problem, defined above, is that there is no need to derive explicit mathematical relationships, because networks, during the machine learning process, ascribe adequate values to the subsequent variables, pursuing a given model, obtained in the process of an experimental research [27,28,29,30]. In the case, presented in the study, the reference values derived from the results of laboratory tests of LWC, containing granulated expanded glass aggregate (GEGA) and granulated ash aggregate (GAA) [31,32,33,34]. The present study reveals the possibility of using artificial neural networks to predict the parameters of LWC e.g., strength, porosity, and apparent density.

An additional purpose of the work was to check existing computer techniques in the form of ANNs, which can be used to design the LWC composition with the desired properties. 

## 2. Materials and Methods 

Portland cement CEM I 42,5R according to [35] was used to perform the tests. Chemical composition and physical properties of the cement CEM I 42,5R were shown in Table 1. 

**Figure 1:**
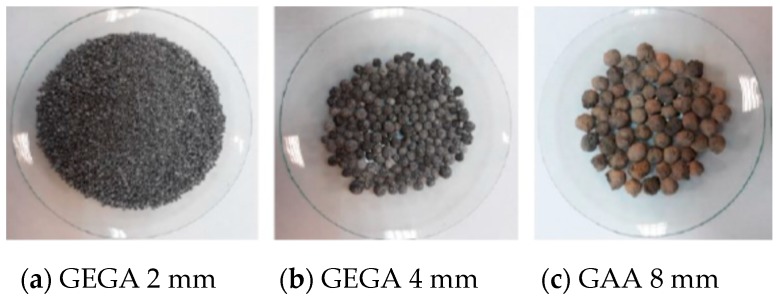
Aggregates: (**a**,**b**) granulated expanded glass aggregate (GEGA), (**c**) granulated ash aggregate (GAA).

GEGA of 2 and 4 mm grain size (Figure 1a,b) and GAA of 8 mm grain size (Figure 1c) were used as the main component of LWC. Chemical composition of aggregates was given in Table 2. 

The main component of cement is CaO, and its content is from 4% to 63%, while the content of SiO_2_ silica is approximately 21.7%. It is shown in Table 1. The main component of LWA (GEGA and GAA) is SiO_2_ silica, and its content in GEGA is from 33% to 63% and in GAA is 52.82%, as shown in Table 2. Physical properties of the aggregates are presented in Table 3.

The purpose of laboratory tests was to determine the influence of the type, grain size and percentage of content of lightweight aggregates on physical and mechanical properties of the concrete. The mixture of the following composition was designed: CEM I 42,5R–500 kg, all batch water (free water and moisture in aggregates)–250 kg. The tested aggregate was not pre-moisturised in the laboratory, it contained some amount of natural moisture, which was measured just before mixing. It was as follows: GEGA 2 mm–3.4%, GEGA 4 mm–2.9%, GAA 8 mm–4.1%. Water to cement ratio was w/c = 0.5. No chemical admixtures or additives were used in the test. Constant volume of the aggregate of 550 dm^3^ was adopted, preliminary assuming the porosity of LWC at approximately 10%. There were 15 versions of LWC mixtures designed, marked LWC 1-15, of different percentage shares of GEGA 2 mm, GEGA 4 mm, GAA 8 mm aggregates. Percentage share of each aggregate was designed at the range of 0%, 25%, 50%, 75% and 100% and it is presented in Table 4. 

The components of the mixture were mixed in a mechanical mixer. First, cement and water were mixed for 2 min. Then, the aggregate was added to the slurry in accordance with the designed composition of LWC 1-15 and mixed for another 2 min. There was 30 min allocated from the first contact of the cement with water. Consistency was tested by means of the slump method in accordance with [36]. Consistency of the mixtures LWC 1–4 and LWC 6–13 in accordance with [37] were within the range of S3 (100–150 mm), while the one of LWC5 and LWC 14 and LWC 15 were within the range of S2 (50–90 mm). Concrete mixture was laid in PVC moulds in accordance with [38] in two layers and vibrated on a vibration table in accordance with [39]. Compaction of GEGA concrete mixture had to be carried out so as not to damage the grains of the aggregate. Six samples for each LWC 1-15 variant were made of 15 × 15 × 15 cm and were tested for compressive strength on the 28th day, while 3 cubic specimens of 10 × 10 × 10 cm for each composition variant of aggregates were made for the test of apparent density and porosity of the concrete. Test specimens were stored for 24 h in a mould, in the temperature of 20 ± 2 °C, followed by subsequent storage in the chamber with the humidity of 95–100% and the temperature of 20 ± 2 °C and protected against drying in accordance with [40]. On the 28th day, 1 h before the test, specimens were taken out from the chamber and left to dry in the air, in the temperature of 20 ± 2 °C. 

Hydrostatic method (called also Archimedes method) was used for density and porosity testing. Each result of porosity test is an arithmetic mean of three independent measurements.

Compressive strength of LWC was tested on the 28th day at Controls Advantest 9 machine of maximum pressure force of 3000 kN in accordance with [41]. Compressive strength is an average value of six results obtained. 

## 3. Results

Mechanical properties of the concrete with GEGA and GAA were presented in Table 5, which contains data concerning compressive strength, apparent density and porosity of the tested specimens. 

Table 5 presents the test results of the properties of LWC. Granulated expanded glass aggregate’s (GEGA) features are high porosity, low apparent density and low compressive strength. The analysis of the test results proves that the specimens of composition, designated as LWC 2 and LWC 1 with porosity of 17.7% and 20.8%, respectively, have reached the highest values of compressive strength: 21.35 MPa and 18.65 MPa. The share of GEGA 2 mm aggregate in the LWC 2 concrete was 25% and bulk density of LWC 2 was 1378 kg/m^3^, and it was 12% lower than in the case of the highest density specimen of LWC 1, which was 1560 kg/m^3^. The lowest strength was noted during the test of the specimens, designated as LWC 4, and it was 3.72 MPa. LWC 4 is composed of 75% of GEGA 2 mm and 25% of GAA. Apparent density of LWC 4 concrete was 877 kg/m^3^, it was the lowest measured density in all LWC 1-15. However, the porosity of LWC 4 was 22.1%, the highest one in the obtained results in the range 15.2%–67%. 

The highest volumetric porosity 67% was observed in specimens of LWC 5 concrete with 100% share of GEGA 4 mm, and of LWC 9, with average porosity of LWC 9 was 65.9% (GEGA 2 mm was 25%, GEGA 4 mm was 75%). Average bulk density was, respectively: in LWC 5 was 1078 kg/m^3^, while its compressive strength was 12.49 MPa; and in LWC 9 was 929 kg/m^3^, while compressive strength was 4.21 MPa. The analysis of test results has proved that strength and porosity of aggregate grains significantly influence physical properties of LWC. Comparing test results for the specimens LWC 2 and LWC 13 with similar density, constituting 1378 kg/m^3^ and 1304 kg/m^3^, respectively, it could be observed, that their average compressive strength results are very different and constitute 21.35 MPa and the higher porosity of LWC 13 is the result of applying GEGA with longer diameter and higher porosity. In the case of LWC 2, 25% of GEGA 2 was applied, however, in case of LWC 13, 25% of GEGA 4 was applied. The ratio of GAA in LWC 2 and LWC 13 was the same and constituted 75%.

## 4. Application of ANN

ANN are usually better than analytical methods in cases where the problem of prediction, classification, control or monitoring appears. Their advantage is due to crucial and unique features which is the ability to learn and generalise the acquired knowledge [42,43,44]. The basic feature of ANN as compared to software performing algorithmic data processing is the ability to generalise knowledge for new input data, which has not been known earlier, i.e., non-presented networks during the learning process. This ability of ANN is mathematically defined as the ability to approximate the values of multi-variable functions, as opposed to the interpolation that can be obtained by algorithmic processing. 

Application of ANN to LWC strength analysis is one of the many applications of artificial intelligence methods for modelling the phenomena; analytical description of which is extremely difficult, or even impossible, due to a very large number of parameters needed to describe them and the difficulty of their precise determination and measurement, or the correlations of which are insufficiently accurate. Simulation using ANN (prediction) allows to predict the compressive strength of concrete for any composition of mixture containing GEGA 2 mm, GEGA 4 mm and GAA 8 mm, based on previously obtained laboratory test results. Having analysed the behaviour of ANN on the testing dataset, the solution obtained can be considered accurate enough. Data originating from laboratory tests, subjected to analysis were divided into three datasets: training dataset (70% of the number of cases), testing dataset (15% of the number of cases), and validation dataset (15% of the number of cases). The result of such division of data is that the process of training and correction of ANN’s weights occur on 85% randomly selected input dataset. The validation dataset allows to evaluate the quality of the neural network training process, and the test dataset allows to compare the LWC strength values predicted by the network with the values on which the program was not trained or tested.

Concrete specimens for laboratory tests were created in the way that total amount of GEGA 2 mm, GEGA 4 mm and GAA 8 mm aggregates have always shared for 70% of the specimen’s volume. Individual specimens differed in percentage share of individual aggregates. Since the shares of aggregates have always added up to 100%, it can be considered that the individual specimens were characterised by only two independent variables, e.g., the % share of GEGA 2 mm and the % share of GEGA 4 mm.

The content of GAA 8 mm in a specimen can always be determined as:

(1) share of GAA 8 mm=100% − share of GEGA 2 mm – share of GEGA 4 mm

Thus, it cannot be treated as the third independent variable. 

The ANN, which was built to predict the strength of LWC for the purpose of the discussed problem, has the following topology:

- Characteristics of the problem: regressive; this description of dependencies is used to build models showing the actual relationships between the input data (explanatory ones) and the output variable (explained one). Then, the steps are executed in the following sequence: values of the explanatory variables, AN, then value of the explained variable;
- Number of input data: 15;
- Network type: feedforward ANN with two hidden layers, two input neurons corresponding to the share of GEGA 2 mm and GEGA 4 mm in a specimen, one output neuron corresponding to the predicted value (strength, density or porosity), connections between neurons of “each other” type (full connection);
- Learning algorithm backward error propagation algorithm;
- Number of neurons in the first hidden layer: from 2 to 12, in the second hidden layer from 2 to 17, depending on the forecasted variable;
- Function of error: mean square error of ANN;
- Function of neuron activation–sigmoid function.

In order to study the quality of prediction of LWC properties using ANN, three datasets corresponding to the analysed values such as density, strength, and porosity were created. Out of 15 numerical data from laboratory tests, involving the content of GEGA 2 mm and GEGA 4 mm in the specimen (as independent input variables), and their respective density, strength or porosity of LWC (as an output variable), 13 values were randomly selected, which formed a training dataset for each predicted value. The other two values formed a testing dataset. This procedure was repeated 10 times to obtain 10 different testing datasets. For example, the first training dataset contained the first 13 measurement points, and the corresponding first testing dataset contained the values of the measurement number 14 and 15, the second training dataset contained measurement points excluding measurements number 5 and 9, which were placed in the second testing dataset, etc. The ANN with two hidden layers (approximation of any continuous function is possible, using a single hidden layer network, and for the approximation of a discontinuous function the use of two hidden layers is necessary [45]) was trained by multiple feeding the training dataset to the ANN’s input, until the stabilisation of the total mean square error of the network at a very low level while avoiding the phenomenon of ANN overfitting. 

Next, data from the appropriate testing set were fed to the input of the already trained ANN, and the relative error of ANN was calculated (the sum of the squares of differences between the actual output values (laboratory results) and those, calculated by the network (generated during the prediction), divided by actual output values). This procedure was repeated for the determined architecture of the ANN for each of the 10 previously prepared different training datasets and corresponding testing datasets. This way ANNs of different number of neurons in hidden layers could be compared. The number of neurons in the first hidden layer varied from 2 to 12, and in the second hidden layer from 2 to 17. ANNs with fewer neurons than mentioned above were disqualified, and ANNs with more neurons or larger number of layers did not significantly improve the results, while increasing only the calculation time. The smallest total relative square error for 10 testing datasets during the prediction of density was achieved by the ANN of 2-11-15-1 architecture, i.e., 2 input neurons (shares of GEGA 2 mm and GEGA 4 mm in the specimen), 11 neurons in the first hidden layer, 15 neurons in the second hidden layer and one output neuron (density). In the case of strength and porosity, the best result was achieved by the ANN also of the 2-11-15-1 architecture. 

Table 6 presents the results of density, porosity and strength laboratory tests of LWC for various shares of GEGA 2 mm and GEGA 4 mm in a specimen, and Table 7 presents the results, obtained for the best ANN predicting density, porosity and strength of LWC for 10 testing datasets. 

The comparison of the results of laboratory tests in terms of density, porosity, and strength of LWC with the results, obtained by ANN on testing datasets, presented in the above tables, indicates a high potential and accuracy of the ANN method, which with a good approximation has predicted the unknown analytical dependence of the properties of LWC, depending on the composition of its aggregates.

ANN with weights of connections between neurons, established during the learning process, enable the ability to predict the properties of LWC for the values of parameters not belonging to the training dataset. Moreover, knowing these weights of connections and network architecture, analytical expression, that exactly matches the performance of the network, is received. In the discussed case, it is a non-linear function of two variables (shares of GEGA 2 mm and GEGA 4 mm in the sample), which can be analysed by standard mathematical methods. 

**Figure 2:**
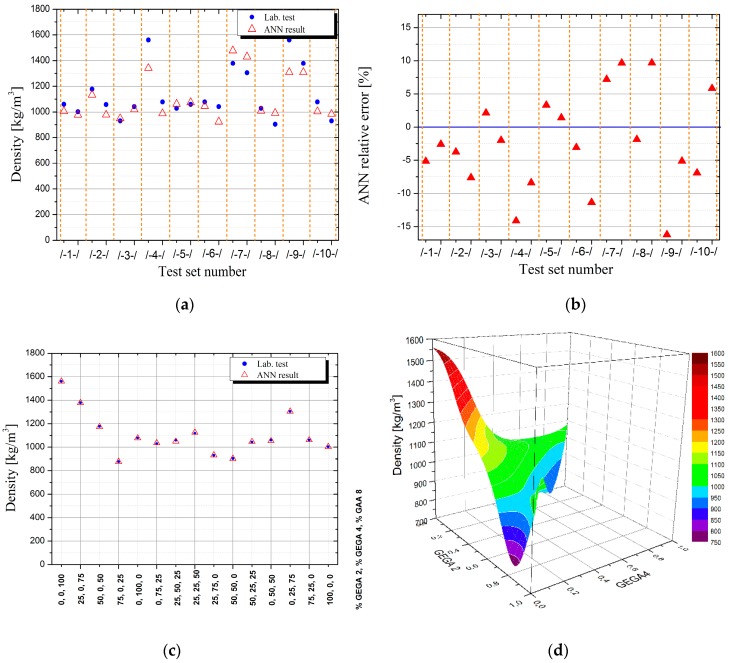
Illustration of results obtained using artificial neural networks (ANN) for density of lightweight concrete (LWC) as a function of GEGA 2 mm and GEGA 4 mm shares in the specimen, (**a**) results generated by ANN for individual testing datasets (**b**) relative error of ANN for individual testing datasets (**c**) comparison of measurement results with values obtained by ANN for training database (**d**) values of density of LWC predicted by ANN for intermediate points between measurement data from the training dataset.

**Figure 3:**
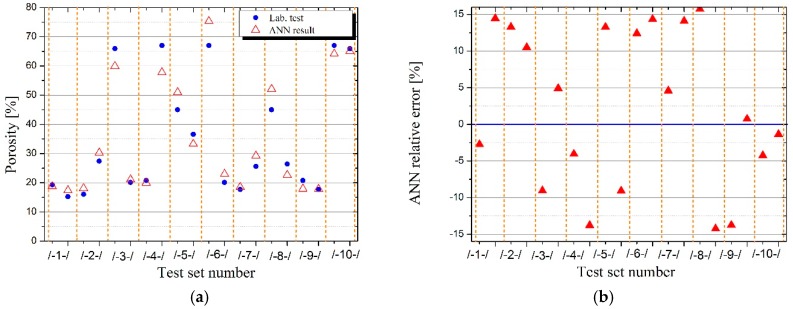

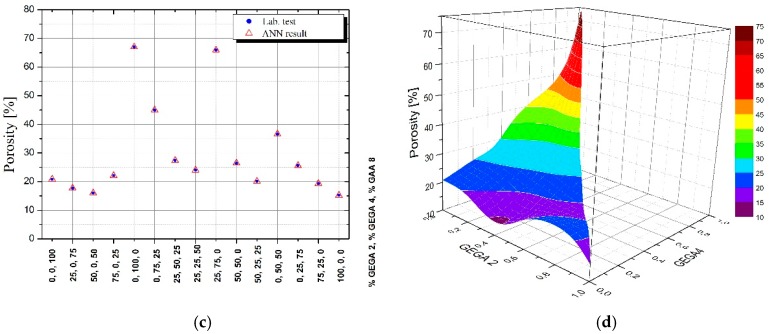
Illustration of the results, obtained by means of using ANN for porosity of LWC as a function of GEGA 2 mm and GEGA 4 mm shares in the specimen, (**a**) results generated by ANN for individual testing datasets (**b**) relative error of ANN for individual testing datasets (**c**) comparison of measurement results with values obtained by ANN for training database, (**d**) values of porosity of LWC predicted by ANN for intermediate points between measurement data from the training dataset.

**Figure 4:**
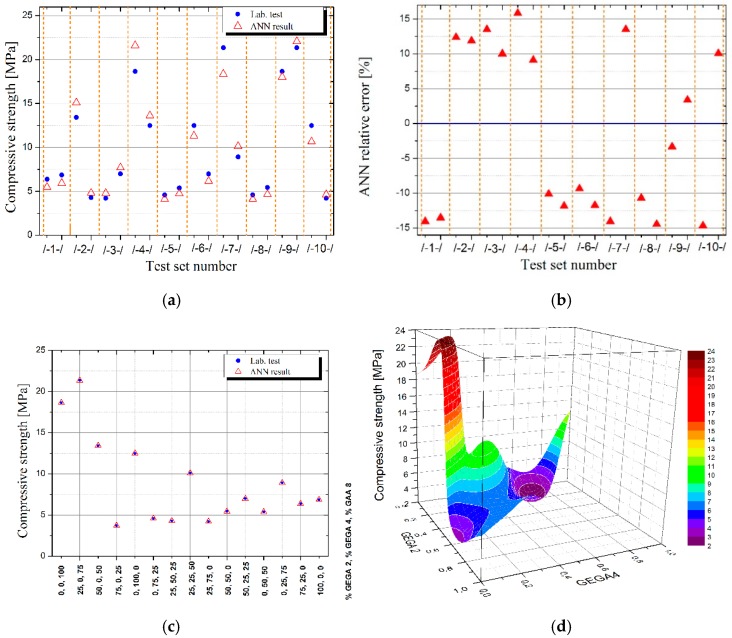
Illustration of the results, obtained by means of using ANN for compressive strength of LWC as a function of GEGA 2 mm and GEGA 4 mm shares in the specimen, (**a**) results generated by ANN for individual testing datasets (**b**) relative error of ANN for individual testing datasets (**c**) comparison of measurement results with values obtained by ANN for training database, (**d**) values of strength of LWC predicted by ANN for intermediate points between measurement data from the training dataset.

In order to illustrate how predicting the properties of LWC by ANN works, input data were prepared for the already learned adequate ANN, in which the shares of GEGA 2 mm and GEGA 4 mm in the specimen changed by e.g., 2% intervals (in measurement data with 25% interval). Results were obtained for the dataset of 1326 measurement points, created in the way, which was illustrated on appropriate 3D charts (Figure 2d, Figure 3d, Figure 4d) and their projections on the plane of variations GEGA 2 mm and GEGA 4 mm (Figure 2a–c, Figure 3a–c, Figure 4a–c). 

The mistakes, generated by ANN during validation (testing) of ANN’s quality on different sets of tests, constitute up to 15%. They are shown in Table 7 and illustrated in Figure 2b, Figure 3b and Figure 4b. They were a result of not only the network’s architecture and properties of the tested function, but also of the fact that ANN, trained on 10 sets of tests was tested in many cases on the variables from outside the set of tests, working in generalization mode. When the values of the variables, used for ANN testing are among the values of the variables, on which ANN was trained, ANN works in function approximation mode. As it is known from ANN theory, the quality of function approximation is much higher than function generalization of the values exceeding the range of ANN training.

**Figure 5:**
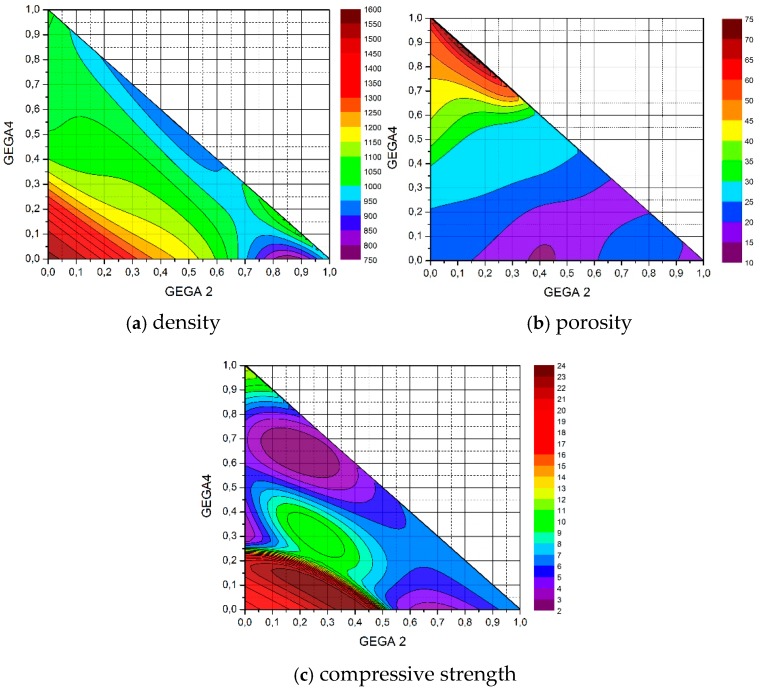
Projections of 3D charts on the plane of the shares of GEGA 2 mm and GEGA 4 mm in a specimen of LWC. Results, generated by ANN (prediction) (**a**) for density, (**b**) for porosity, (**c**) for compressive strength. The charts enable the choice of shares of individual aggregates in the concrete to optimize a few criteria, e.g., the lightest weight concrete of maximum strength.

More specifically describing, the shares of GEGA 2mm and GEGA 4 mm aggregates in the composite, which are independent variables during the ANN training, change from 0 to 1, but their sum must always be less than or equal to 1. Thus, on the variable plane, the problem domain is an isosceles triangle with sides of length 1 (see, e.g., Figure 5). The points corresponding to the laboratory measurements are 15 points from the edge and interior of this triangle, distributed evenly, along the horizontal axis every 0.25 and the vertical axis every 0.25. If, as the ANN training set, we take 13 randomly selected without returning points from the triangle, and the other 2 points used for testing (other than these 13 points), for example, are from the edge of the triangle (for example, points (0,0), (1,0) or (0,1)), the ANN network will generalize the function values beyond the range in which it was trained. If, however, we select a training set such that the points used to test (other than for training) ANN will be from the inside of the triangle (for example points (0.25, 0.25), (0.5, 0.5) (0.25, 75) etc.), then the ANN network it will approximate the function values at these points, which certainly has a smaller error than during the function value generalization.

## 5. Discussion of Results

On the basis of the results of laboratory tests of LWC with GEGA and GAA, it can be concluded, that the kind and the size of the LWA has an enormous influence on LWC properties. Namely, grains porosity and resistance to crushing, significantly influence bulk density, porosity and compressive strength of LWC.

The ratio of the given aggregate in cement composites can be considered a macroscopic parameter, defining the averaged impact of chemical and physical properties of the aggregate on the properties of the composite. GEGA and GAA, applied in research, can be described, using such parameters as presence of the empty spaces inside or outside of them with or without moisture environment, composite concentration in open or closed pores, impact of the aggregates on the composite’s local porosity etc. The above-mentioned parameters can be considered microscopic parameters of different local layouts. The strength of GEGA LWC is advantageously influenced by adding GAA aggregate of higher strength and lower porosity. The highest strength was reached in case of 1:3 ratio of GAA 8 mm to GEGA 2 mm, but the size of GEGA grains also turned out to be important. In case the same ratios of GEGA and GAA shares were used, but the grain size of GEGA was 4 mm, showing that the results were significantly different. 

Analyzing LWC compressive strength tests with GEGA and GAA, it turned out, that the highest compressive strength is being achieved with GAA ratio of 75% and 100% (LWC 1 and LWC 2). The application of 25% of GEGA 2 resulted in about 13% increase in concrete’s compressive strength and about 12% decrease in density and 15% decrease in porosity. 

Assuming that the aggregate with diameter of 2 mm filled the spaces between the aggregates with a diameter of 8 mm, it caused the concrete structure’s sealing, porosity decrease and compressive strength increase. 

Analyzing LWC 5 and LWC 9 research results, it was stated, that when concrete’s bulk density was 15% lower (LWC 9), then, concrete’s compressive strength decreased by 200%. In this case, the application of 25% of GEGA 2 (LWC 9) instead 25% of GEGA 4 (LWC5), which had a major impact on the increase in porosity and drop in compressive strength. However, another phenomenon occurred here, it was observed while preparing and testing the consistency of the concrete mix. Consistency, tested with slump method, was lower in LWC 5 (100% GEGA 4) than in LWC 9 (25% GEGA 2 and 75% GEGA 4). The consistency slump was visible—from 14 to 4 cm. After the test, a part of the grain was crushed in a mechanical mixer and cement paste permeated into GEGA 4. Grain impregnation with cement paste could cause compressive strength’s increase in LWC 5. 

In another case, when concrete density was similar (which concerns LWC 2 and LWC 13), porosity might change by 50%. Porosity increase about 30% resulted in about 200% decrease in LWC compressive strength. The application of GEGA 4 resulted in increase in porosity and decrease in compressive strength. The remaining components remained the same. The use of GEGA with higher porosity and lower compressive strength influenced the final porosity and strength of concrete.

Summarizing the analysis, it may be observed, that the highest compressive strength can be reached by LWC specimens, containing 50%, 75% and 100% share of GAA 8 mm and GEGA 2 mm maximum 25%. Lower results of strength tests were noted for the specimens, containing the aggregate of the highest porosity and the lowest compressive strength when used 50%, 75% or 100% GEGA 4 mm. It can be concluded, that the content of aggregate with grain size diameter longer than 2 mm will influence the strength of LWC, and the higher the share of the aggregate is, the higher porosity and the lower the specimen’s compressive strength is.

The results, shown in Table 6 form the dataset, which were used to train and test ANN. Typically, from this dataset from 5 to 15% of elements were randomly selected to form a test dataset for ANN. The other elements constituted the training dataset for ANN. The training dataset in our research contained 13 elements, and the test dataset contained 2 elements. Usually, available data are randomly divided only once into a training and test dataset. Instead, to increase the quality of ANN in this study, we have applied a cross-validation procedure. 

Therefore, 10 variants of different data for training and checking the performance of ANN were prepared. For example, the first training dataset contained the first 13 measurement points, and the corresponding first testing dataset contained the values of the measurement number 14 and 15, the second testing training dataset contained measurement points, excluding measurements number 5 and 9, which were placed in the second testing dataset, etc. For each of the 10 cases, ANN was trained on a 13-element dataset and tested on a 2-element dataset that was not used during the ANN training. In each case, ANN network had different weights between neurons (the architecture of ANN was fixed). This procedure was repeated for different ANN architectures, i.e., for different numbers of neurons in the first and second hidden layer. By analyzing the total mean square error of each ANN, the best ANN was selected. What is important was that ANN was tested every time on a dataset that was not used during the ANN training. Good quality of ANN forecasting in this case is related to the fact, that the training dataset had relatively few elements and that the dependence of predicted LWC properties is a smooth function of GEGA 2 mm, GEGA 4 mm aggregate shares. 

To give it a more detailed description, the ratios of GEGA 2 mm and GEGA 4 mm in the composite, which become independent variables during ANN training, change from 0 to 1 (from 0% to 100%), but their sum must always either equal 1 or be less than 1. When their sum equals 1, it means a lack of GAA 8 mm in the composite; when their sum is less than 1, then, this missing value, needed to constitute 1, means the presence of GAA 8 mm. Therefore, considering variables, we need to bring about the issue of isosceles triangle with side length of 1 (see Figure 5). 

With a trained ANN, recorded in the form of an analytical formula or 3D charts, generated by it, it is possible to optimize the composition of aggregates in LWC in order to obtain its desired properties (Figure 5a,b). For example, to suggest LWC composition of maximum compressive strength for a given range of its density or porosity (Figure 5c).

As the figure shows, the ratio of GAA 8 mm has the greatest influence on the density of the composite. The greatest density values are located around the point (0,0), which means, those with a low ratio of GEGA 2 mm and GEGA 4 mm. The lowest density values are obtained, when the ratio of GEGA 2 mm prevails in the composite. The greatest porosity of the composite is obtained, when the ratio of GEGA 2 mm and GEGA 4 mm prevail over the ratio of GAA 8 mm. Taking into account ANN testing process, porosity values may be insignificantly higher in the graph. The greatest compressive strength is obtained, when the ratio of GEGA 2 mm and GEGA 4 mm is about 35% and the rest (about 65%) is constituted by GAA 8 mm. The lightest composites and the most porous composites are characterized by low compression strength.

On the basis of the analysis, supplied in this paper, it can be concluded that ANN is a useful tool for designing parameters for the new laboratory tests, saving time, and costs of production of the specimens of composites, which can show undesirable properties during testing.

## 6. Conclusions

The results of the presented research show the new possibilities of the application of computer tools in concrete technology. In the currently used solutions for LWC, additional benefits are developed, e.g., improvement of insulating properties with simultaneous increase of load-bearing capacity, extension of durability, or improvement of surface aesthetics [46,47,48,49,50,51,52,53]. It should be emphasized that these computer tests with the use ANN were preceded by theoretical considerations and long laboratory tests. Practical insights are extremely important to fully understand the process of designing the structure of LWC [54,55,56]. For this reason, the superiority of analytical over experimental methods only should be emphasized. 

In order to enable any theoretical model (mathematical, geometric) to be used in practical design of concrete mix composition, it must be properly calibrated. Calibration is also necessary in case of each change of input parameters, as well as when new variables are introduced. This process requires necessity of concrete laboratory tests. Laboratory testing usually requires a certain amount of time and is expensive. The given study presents the case of predicting performance of LWC containing granulated expanded glass aggregate and granulated ash aggregate by means of using ANNs. In the course of examining the problem, the values, close to the experimentally determined strength, were obtained. The advantage of the applied methodology is that many additional parameters can be introduced into the concrete mixture design, and the ANN will continue to learn the relationships between the individual data and verify their impact on the final result in the specified range. It was proven in the analysed case that from the point of view of the global sensitivity analysis, the most important variables are: percentage share and proportions of the used lightweight aggregate. 

Based on this study, it can be concluded that the presented case of ANN application for predicting compressive strength of LWC may be useful for the development of analytical and experimental methods of designing the composition of LWCs and other cement composites. Furthermore, such a solution can have a significant impact on the verification and calibration of existing methods and models and also, supported by a sufficiently large set of experimental data, it can be used to create a virtual laboratory for technology engineers. 

The tests results and their analysis allow to observe the benefits of ANN application for LWC properties prediction in case the components’ properties are known. It also enables to design a certain composition of LWC with the desired properties. The possibility of application of other components than those, described in this paper, has to be assessed in further research. An important aspect, worth considering in further work, is looking into the effects of the use of different kinds of cement, additives and aggregates, allowing to design the composition of LWC with, for instance, the lowest bulk density, high compression strength and with the lowest thermal conductivity coefficient.

## Figures and Tables

**Table 1:** Chemical composition and physical properties of cement CEM I 42,5R [15].

Setting Start Time [min]	Setting End Time [min]		Compressive Strength [MPa]			Blaine Fineness [cm^2^/g]		Loss of Roasting [%]			Water Demand [%]	
			2d		28d							
155	195		30.2		57.3	3504		3.4			27.5	
**Content [%]**												
SiO_2_	Al_2_O_3_		Fe_2_O_3_	CaO	MgO	SO_3_	Na_2_O		K_2_O	TiO_2_		Cl
21.7	6.2		3.1	63.4	1.0	3.9	0.16		0.64	0.25		0.06
**Mineralogical composition, content [%]**												
Na_2_Oeq		C_3_S			C_2_S	C_3_A			C_4_AF			
0.7		63.1			7.6	6.1			8.9			

**Table 2:** The chemical composition of the aggregate [15].

Aggregate Type	Content [%]								
	SiO_2_	Al_2_O_3_	Fe_2_O_3_	CaO	MgO	SO_3_	Na_2_O	K_2_O	Loss of Roasting
**GEGA**	63.33	0.74	-	14.19	2.98	0.32	13.35	0.57	4.53
**GAA**	52.82	24.28	7.5	4.5	3.19	0.43	-	0.2	7.1

**Table 3:** Physical properties of the aggregates [15].

Property		GEGA 2 mm	GEGA 4 mm	GAA 8 mm
Water absorption WA_24_	[%]	15.2	17.8	16.5
Volume density ρ_a_	[kg/m^3^]	380	350	1350
Density of dried grain ρ_rd_	[kg/m^3^]	340	310	1250
Density of saturated grain ρ_ssd_	[kg/m^3^]	360	330	1290
Porosity P	[%]	37	42	37
Crumble indicator *X_r_*	[%]	22.3	25.9	17.8
pH after 24 h	-	11.9	11.9	11.1
Bulk density in a loose state *ρ_b_*	[kg/m^3^]	200	180	680
Thermal conductivity of 40 cm layer of aggregate	W/m·K	0.71	0.69	0.85

**Table 4:** Percentage share of the aggregates.

	Specimens Designation [%]														
LWC	1	2	3	4	5	6	7	8	9	10	11	12	13	14	15
GEGA 2 mm	0	25	50	75	0	0	25	25	25	50	50	0	0	75	100
GEGA 4 mm	0	0	0	0	100	75	50	25	75	50	25	50	25	25	0
GAA 8 mm	100	75	50	25	0	25	25	50	0	0	25	50	75	0	0

**Table 5:** Test results for lightweight concrete (LWC).

	Specimens Designation LWC														
	1	2	3	4	5	6	7	8	9	10	11	12	13	14	15
Apparent density [kg/m^3^]	1560	1378	1177	877	1078	1028	1058	1117	929	903	1041	1059	1304	1060	1002
Porosity *p_o_*[%]	20.8	17.7	16.0	22.1	67	45.0	27.4	24.0	65.9	26.4	20.1	36.6	25.6	19.3	15.2
Compressive strength [MPa]	18.65	21.35	13.43	3.72	12.49	4.59	4.29	10.1	4.21	5.44	6.99	5.38	8.92	6.37	6.86

**Table 6:** Results of laboratory tests.

Lab, Test LWC	Share of GEGA 2 mm	Share of GEGA 4 mm	Concrete Density [kg/m^3^]	Concrete Porosity [%]	Concrete Strength [MPa]
1	0	0	1560	20.8	18.65
2	0.25	0	1378	17.7	21.35
3	0.5	0	1177	16.0	13.43
4	0.75	0	877	22.1	3.72
5	0	1.0	1078	67.0	12.49
6	0	0.75	1028	45.0	4.59
7	0.25	0.50	1058	27.4	4.29
8	0.25	0.25	1117	24.0	10.1
9	0.25	0.75	929	65.9	4.21
10	0.5	0.5	903	26.4	5.44
11	0.5	0.25	1041	20.1	6.99
12	0	0.5	1059	36.6	5.38
13	0	0.25	1304	25.6	8.92
14	0.75	0.25	1060	19.3	6.37
15	1.0	0	1002	15.2	6.86

**Table 7:** Results predicted by the artificial neural networks (ANN) during the validation procedure.

Testing Dataset	Network 2-11-15-1 for Density			Network 2-11-15-1 for Porosity			Network 2-11-15-1 for Strength		
	Lab. Test	ANN	Relative Error [%]	Lab. Test	ANN	Relative Error [%]	Lab. Test	ANN	Relative Error [%]
1 dataset (Lab. test LWC 14,15)	1060	1005.31	−5.16	19.03	18.773	−2.73	6.37	5.476	−14.04
	1002	976.03	−2.59	15.2	17.397	−14.45	6.86	5.933	−13.52
2 dataset (Lab. test LWC 3,7)	1177	1132.83	−3.75	16.0	18.126	13.28	13.43	15.091	12.37
	1058	977.27	−7.63	27.4	30.276	10.49	4.29	4.799	11.86
3 dataset (Lab. test LWC 9,11)	929	948.78	2.13	65.9	59.937	−9.05	4.21	4.778	13.49
	1041	1020.21	−1.99	20.1	21.086	4.91	6.99	7.689	10.01
4 dataset (Lab. test LWC 1,5)	1560	1339.91	−14.11	20.8	19.959	−4.04	18.65	21.601	15.82
	1078	987.66	−8.38	67.0	57.772	−13.77	12.49	13.627	9.11
5 dataset (Lab. test LWC 6,12)	1028	1062.04	3.31	45.0	50.97	13.27	4.59	4.126	−10.11
	1059	1073.93	1.41	36.6	33.278	−9.08	5.38	4.743	−11.84
6 dataset (Lab. test LWC 5,11)	1078	1044.83	−3.08	67.0	75.317	12.41	12.49	11.322	−9.36
	1041	923.08	−11.33	20.1	22.982	14.34	6.99	6.169	−11.75
7 dataset (Lab. test LWC 2,13)	1378	1477.18	7.19	17.7	18.512	4.59	21.35	18.351	−14.05
	1304	1429.97	9.66	25.6	29.21	14.1	8.92	10.125	13.51
8 dataset (Lab. test LWC 6,10)	1028	1008.79	−1.87	45.0	52.077	15.73	4.59	4.099	−10.71
	903	990.43	9.68	26.4	22.641	−14.24	5.44	4.654	−14.46
9 dataset (Lab. test LWC 1,2)	1560	1307.31	−16.19	20.8	17.937	−13.76	18.65	18.026	−3.35
	1378	1305.18	−5.13	17.7	17.829	0.73	21.35	22.068	3.36
10 dataset (Lab. test LWC 5,9)	1078	1003.46	−6.91	67.0	64.153	−4.25	12.49	10.658	−14.67
	929	983.25	5.84	65.9	65.008	−1.35	4.21	4.633	10.05

## Shortcuts

LWC	lightweight concrete
LWA	lightweight aggregate
GEGA	granulated expanded glass aggregate
GAA	granulated ash aggregate
ANN	artificial neural networks
